# Cost of treating sick young infants (0-59 days) with Possible Serious Bacterial Infection in resource-constrained outpatient primary care facilities: An insight from implementation research in two districts of Haryana and Uttar Pradesh (India)

**DOI:** 10.7189/jogh.13.04062

**Published:** 2023-08-18

**Authors:** Charu C Garg, Rupak Mukopadhyay, Narendra Kumar Arora, Shally Awasthi, Raj Kumar Verma, Ramesh Poluru, Priya Limbu, Shamim Ahmad Qazi, Rajiv Bahl, Yasir Bin Nisar

**Affiliations:** 1 Health Financing Advisor and Executive Director, Syzygy Consulting, California, USA; 2 Centre for Anthropology, Amity University, Uttar Pradesh, Noida Campus, India; 3 The INCLEN Trust International, New Delhi, India; 4 Department of Paediatrics, King George's Medical University (KGMU), Lucknow, India; 5 The George Institute of Global Health, New Delhi, India; 6 Newborn and Child Health Consultant, Geneva, Switzerland; 7 Indian Council of Medical Research, New Delhi, India; 8 Department of Maternal, Newborn, Child and Adolescent Health and Aging (MCH), World Health Organization, Geneva, Switzerland

## Abstract

**Background:**

Information on the average and incremental costs of implementing alternative strategies for treating young infants 0-59 days old in primary health facilities with signs of possible serious bacterial infection (PSBI) when a referral is not feasible is limited but valuable for policymakers.

**Methods:**

Direct activity costs were calculated for outpatient treatment of PSBI and pneumonia in two districts of India: Palwal, Haryana and Lucknow, Uttar Pradesh. These included costs of staff time and consumables for initial assessment, classification, and referrals; recommended treatment of fast breathing (oral amoxicillin for seven days) and PSBI (injection gentamicin and oral amoxicillin for seven days); and daily assessments. Indirect operational costs included staff training; staff time cost for general management, supervision, and coordination; referral transport; and communication.

**Results:**

The average cost per young infant treated for recommended and acceptable treatment for PSBI was 16 US dollars (US$) (95% CI = US$15.4-16.3) in 2018-19 and US$18.5 in 2022 (adjusted for inflation) when all direct and indirect operational costs were considered. The average cost of recommended treatment for pneumonia was US$10.1 (95% CI = US$9.7-10.6) or US$11.7 in 2022, per treated young infant. The incremental cost 2018-2019 for supplies, medicines, and operations (excluding staff time costs) per infant treated for PSBI was US$6.1 and US$4.3 and for pneumonia was US$3.5 and US$2.2 in Palwal and Lucknow, respectively. Operation and administrative costs were 25% in Palwal and 12% in Lucknow of the total PSBI treatment costs. The average cost per live birth for treating PSBI in each population was US$5 in Palwal and US$3 in Lucknow. Higher operation costs for social mobilisation activities in Palwal led to the empowerment of families and timely care-seeking.

**Conclusions:**

Costs of treatment of PSBI with the recommended regimen in an outpatient setting, when a referral is not feasible, are under US$20 per treated child and must be budgeted to reduce deaths from neonatal sepsis. The investment must be made in activities that lead to successful identification, prompt care seeking, timely initiation of treatment and follow-up.

The incidence of neonatal sepsis in India is the highest in the world and is the second leading cause of neonatal mortality in India [1,2]. With 17 deaths per 1000 live births globally in 2020 [3], the neonatal mortality rate of 24.9 per thousand reported in 2019-21 in India, is high [4]. The World Health Organization (WHO) recommends referral to hospital and injectable therapy for the management of sepsis / possible serious bacterial infection (PSBI) in young infants (0-59 days old) [5,6]. However, in resource-limited settings, a large proportion of the families of young infants with signs of severe infection do not accept a referral to a hospital due to various reasons, such as distance to the health facility, cost of hospitalisation, daily wage loss during hospitalisation period and cultural constraints resulting in newborn deaths [7,8].

Randomised controlled trials conducted in Africa and Asia demonstrated that simplified antibiotic regimens given to young infants with PSBI in outpatient settings when a referral is not feasible were effective [9-13]. To increase access to treatment, WHO used the evidence to strategise a guideline to manage young infants with PSBI on an outpatient basis when a referral is not feasible [6]. The Government of India accordingly harmonised the integrated management of neonatal and childhood illness (IMNCI) protocols for providers [14-16]. While these guidelines provide an important framework to identify, assess, classify, and treat young infants with PSBI in outpatient settings, there have been barriers to effective implementation [17-21].

Data about the costs of treatment for the management of neonatal infections, especially in low-resource settings, is quite limited. The information on the cost of making such guidelines operational is limited too. A study from Ethiopia assessed the costs of managing PSBI for young infants at health posts when a referral was not possible and found it cost-effective [22]. A systematic review from both low- and middle-income countries (LMIC) and high-income countries (HIC) reported direct costs for treatment for pneumonia in community, outpatient and inpatient settings [23]. The African Neonatal Sepsis Trial (AFRINEST) collected costs for the management of PSBI with simplified antibiotic regimens in the Democratic Republic of Congo, Kenya and Nigeria and found them cost-effective [24].

Recently, multi-centric implementation research was undertaken at four districts in India to contextualise strategies to address implementation bottlenecks of the national PSBI Management Guideline (when a referral is not feasible) and strengthen its on-ground implementation of treatment in outpatient primary health care settings [18-21]. At two of the sites – Palwal, Haryana and Lucknow, Uttar Pradesh [18,19], we estimated the average total and incremental costs of these interventions for outpatient treatment of PSBI with simplified antibiotic regimens, reported here. This study also identifies the costs for specific interventions that are important in removing the implementation bottlenecks for the management of young infants with PSBI when a referral is not feasible.

## METHODS

The costing data was collected from two PSBI implementation research sites (Lucknow district of Uttar Pradesh and Palwal district of Haryana) [18,19], which were part of multi-centric implementation research between June 2017 to February 2019 [18-21]. The cost data were collected from March 2018 to February 2019. These sites were mainly rural, with some semi-urban and peri-urban areas. Details of the implementation methodology have been reported elsewhere [18,19].

### Framework for analysis

The framework for determining the cost of PSBI management was conceived following national program guidelines [14-16] and the interventions that were identified based on the experiences and observations made during the implementation studies [18,19]. As the implementation of PSBI management guidelines is embedded in three national programs, i.e., Janani Suraksha Yojna (JSY) [25], Home-based Newborn care (HBNC) [26], and Integrated Management of Neonatal and Childhood Illnesses (IMNCI) [27], the existing costs that were already budgeted under these programs, such as printing cost for data collection and reporting formats, were not included or only partially included. The costs were estimated for resources that were additionally invested to successfully implement the different components of the PSBI guideline. The costs were estimated for two dimensions: 1) direct intervention / activity costs, and 2) indirect program operational costs.

### Direct intervention / activity cost

These included costs of staff’s additional time, medicine, and essential supplies for strengthening activities that were directed to each mother-child in outpatient settings. The intervention cost framework had three core domains.

#### Domain1: Identification and referral / mobilisation of sick young infants at the community level

##### Postnatal home visits for identifying danger signs

Six home visits for facility-based birth and seven postnatal visits for home-based births were made by the Accredited Social Health Activist (ASHA) for birth identification, measuring young infant’s weight, promoting optimal care practices such as breastfeeding, keeping the baby warm, promote hygiene, recognise “danger signs” in mothers and newborns and promote appropriate and prompt care seeking. Mothers were given cards to identify danger signs.

##### Mobilisation of sick young infants to care provider / health facility

Once the ASHA identified any danger signs at the community level, young infants were referred / mobilised to a higher-level facility for further assessment, generally a primary health centre (PHC) or community health centre (CHC), but rarely a subcenter. Families usually travelled to health facilities by themselves, however, in some instances, they were accompanied by ASHAs and occasionally used government ambulances for transportation. ASHA also revisited the homes to check the outcome of the referral or treatment.

#### Domain 2: Management of sick young infants at the facility level using IMNCI protocols

##### Assessment and classification

Once sick young infants were presented in health facilities, the IMNCI-trained Auxiliary Nurse Midwife (ANM) or nurse or a Medical Officer (MO) assessed and classified them as per the IMNCI algorithm [14]. Those with a minor local infection, jaundice, dehydration, or feeding problem were given recommended medicines / treatment and sent home. The cost of treating minor ailments is not included as these are not part of PSBI treatment. Those young infants 7-59 days old with fast breathing only (60 breaths or more per minute) were classified as pneumonia, and others with any sign of PSBI were reclassified into three PSBI categories – 1) severe pneumonia (young infants <7 days old with fast breathing only), 2) clinical severe infection (stopped feeding well, body temperature 38°C or more or less than 35.5°C, severe chest indrawing, and movement only when stimulated), 3) critical illness (convulsions, no movement at all, not able to feed at all).

##### Counselling for those with PSBI for referral to higher health facilities

All young infants who had any sign of PSBI were referred to a higher-level facility (generally district hospitals) for further treatment as per guidelines. Staff time costs for assessment and counselling on referral and care during referral transportation were included.

##### Pre-referral treatment

Those who accepted referrals were given a referral form / slips, and a pre-referral dose of injection gentamicin and oral amoxicillin. The costs of both were included under supplies.

##### Treatment for pneumonia

Young infants 7-59 days old with fast breathing (60 breaths or more per minute) were treated as outpatients in a subcenter, PHC or CHC with oral amoxicillin. Mothers were taught to administer the dosage twice daily at home for seven days.

#### Domain 3: Outpatient treatment for PSBI in primary care settings when hospitalisation was not feasible

The families, who did not accept the referral, were treated at a primary health facility on an ambulatory basis following national guidelines. Treatment of these sick young infants was based on re-classification into the following three categories: 1) young infants zero-six days old with severe pneumonia (60 breaths or more per minute) were treated with intra-muscular (IM) gentamicin (once daily) and oral amoxicillin (twice daily for seven days); 2) infants with any sign of clinical severe infection were treated with daily IM gentamicin and twice daily oral amoxicillin for seven days; 3) infants with PSBI critical illness were to be treated with a daily dose of IM gentamicin and twice daily IM ampicillin. Under each PSBI subclassification, costs of staff time to provide treatment, costs for medicines and consumable supplies such as syringes, needles, methylated spirit, and a cotton swab were calculated.

While Lucknow had follow-up visits on days four and seven after the initiation of outpatient treatment, Haryana reported that mothers were asked to return to the facility on the fourth day of treatment for pneumonia, whereas for PSBI, the follow-up visits happened on all days infants were brought in for gentamicin injection. Staff costs were calculated for time spent on follow-up and the number of visits reported at each site. The follow-up visit was not differentiated by the type of treatment received.

The treatments received by sick young infants were classified as recommended, acceptable, partial, and non-compliant, based on the number of days and types of medicines given to them (**Table 1**). We noted that for non-compliant treatment, different medications were given, such as 1) cefixime in Palwal and cefpodoxime + clavulanic acid in Lucknow, or 2) non-antibiotics such as paracetamol, cetirizine, gentian violet with or without gentamicin and amoxicillin. We estimated the costs of only recommended and acceptable treatment for PSBI and pneumonia. We included only clinical severe infections and severe pneumonia under PSBI as injection ampicillin required for critical illness was unavailable at PHC and CHCs, and therefore the infants with critical illness who did not accept referral were treated with non-recommended treatment only.

**Table 1:** Various categories of treatments received by sick young infants as outpatients when a referral is not accepted

No	Types of treatment	PSBI: clinical severe infection and severe pneumonia	Pneumonia / fast breathing
1	Recommended treatment	7 d of injection gentamicin (once daily) and 7 d of oral amoxicillin (twice daily)	7 d of oral amoxicillin (twice daily, a total of 14 doses)
2	Acceptable treatment	2-6 d of injection gentamicin (once daily and 7 d of oral amoxicillin (twice daily)	Between 10 to 13 doses of oral amoxicillin
3	Partial treatment*	0-1 d of injection gentamicin (once daily) and 7 d of oral amoxicillin (twice daily); any days of injection gentamicin (even 7) and less than 7 d of oral amoxicillin (twice daily)	<10 doses of oral amoxicillin
4	Non-compliant treatment / non-recommended treatment	Other antibiotics in a combination of injection gentamicin and oral amoxicillin; other antibiotics used exclusively; use of antipyretic / other non-antibiotics exclusively	Another antibiotic in combination with oral amoxicillin; other antibiotics used exclusively; use of antipyretic / other non-antibiotics exclusively

PSBI – possible serious bacterial infection, d – days

*Partial treatment was mainly due to the family's perception of improvement in the young infant’s condition. Other reasons were the inconvenience to pay a daily visit, discomfort in accepting daily injections for the newborn, or the infant dying before the completion of treatment.

The items included for costing under the three core domains of the intervention framework for recommended and acceptable treatment are summarised in **Table 2**.

**Table 2:** Cost items included under three core domains of the intervention framework for recommended / acceptable treatment

No	Interventions / activities	Staff / provider's costs	Supplies costs
**Domain 1**	**Identification and referral / mobilisation of sick young infants at the community level**		
1.1	Postnatal home visits for identifying danger signs	20% of the incentives of ₹250* to ASHA for infants receiving 6-7 post-natal visits and ₹125 to those receiving 1-5 visits	Mother’s card for danger signs identification
1.2	Referral / mobilisation to the nearest health facility for treatment	ASHA time cost is not included but transport incentives (₹100) were given to ASHA for all PSBI-referred children	PSBI referral and follow-up slip (only used in Palwal)
**Domain 2**	**Management of sick young infants at the facility level using IMNCI§ protocols**		
2.1	Assessment and classification	ANM / nurse / medical officers’ time cost per child assessed	Not applicable
2.2	Counselling and referral advice to higher facilities for PSBI cases	ANM / nurse / medical officers’ time cost per child	Not applicable
2.3	Pre-referral dose for those who accepted a referral	Nurses’ / medical officers’ time for filling out a referral form and giving the pre-referral dose	Cost of the referral form and one pre-referral dose of gentamicin and amoxicillin
2.4	Treatment of infants 7-59 d with fast breathing (pneumonia)	ANM / nurse and / or medical officers’ time cost per infant treated	7 d of oral amoxicillin (twice daily, a total of 14 doses) or 10-13 doses over oral amoxicillin
**Domain 3**	**Outpatient treatment for PSBI in primary care settings when hospitalisation was not feasible**		
3.1	Treatment of infants 0-6 d with fast breathing (severe pneumonia)	ANM / nurse and / or medical officers’ time cost per infant treated	7 d of injection gentamicin (once daily) and 7 d of oral amoxicillin (twice daily) or 2 - 6 d of injection gentamicin (once daily and 7 d of oral amoxicillin (twice daily). Linked syringe, needle, spirit, and cotton swab
3.2	Treatment of infants with clinical severe infection	ANM / nurse and / or medical officers’ time cost per infant treated	Same as 3.1
3.3	Follow-up facility visit by the sick young infant for reassessment	ANM / nurse and / or medical officers’ time cost per infant treated	Not applicable

d – days, ASHA – accredited social health activist, PSBI – possible serious bacterial infection, IMNCI – integrated management of newborn and childhood illness, ANM – auxiliary nurse midwife

*Market exchange rate for July 2018 was 1 US dollar (US$) = Indian Rupee (₹) 68.5.

### Indirect or operational cost

These included services and goods that were not specific for the infant treated but were used for activities which could benefit all newborns and were necessary for effective guideline implementation.

### Staff administrative costs

This cost was calculated for the staff time required for strategic and general management (governance level) 1) coordination and supervision by senior medical officers such as handholding and confidence building for medical officers and nurses at PHC and CHC 2) for ASHA coordinators, ANM, lady health visitors (LHV) at facility and field level.

### Training costs

Recurrent costs of training for an average of one to four days were included for different providers at each site. The training of trainers was considered a startup cost and not included in annual recurrent costs.

### Other operational costs

These include 1) demand generation activities, including communication and community mobilisation activities such as organising school rallies and counselling sessions at Village Health and Nutrition Day (VHND), along with the cost for information, education, and communication (IEC) materials like counselling cards, wall painting, banners, leaflets, danger sign cards for mothers and 2) job aids such as training manual, and treatment displays. Research and most health system costs were not included. Costs required for implementation research such as the development of research instruments (data collection formats), study protocol development, translation of PSBI management guidelines into the local language (Hindi), initial training of the research staff, staff hired to monitor the effectiveness of the antibiotic regimen; costs of baseline surveys, workshops, were not included. Infrastructure and equipment costs for health systems such as power, computing / office, transport, and communication equipment; data management, reporting and logistics; non-consumable items such as thermometers and timers were also not included. Fifty percent of the costs of HBNC kits and weighing scales were included as these were considered important for strengthening correct weighing and identifying danger signs.

### Data

Data were collected from each site on the types and the total number of staff involved in PSBI activities and their average annual salaries; the number of live births; the number of young infants receiving one-seven postnatal visits; and the number of infants referred, assessed, and treated under different activities. The total number of treated young infants was estimated by adding all those who received recommended or acceptable treatment for PSBI or pneumonia. If an infant was likely to get more than one visit for an activity such as postnatal visits, then the data was compiled for the number of visits for that intervention / activity, otherwise was compiled on the number of infants receiving that intervention.

We also collected data on time duration (in minutes) taken by the providers for each activity, using structured interview schedules from medical officers, nurses and ANMs working at sub-centres, PHC and CHC. We collected the data using two approaches: 1) third-party observation – where the researcher directly observed the time taken for each sub-activity, and 2) self-observed / self-reported method – wherein the health providers noted the timing by themselves for activities undertaken [28]. The number of providers that participated in this survey at each type of facility is elaborated in the Table S1 in the **Online Supplementary Document**. The average salaries of each provider type were also collected, as shown in the Table S2 in the **Online Supplementary Document**.

Data were collected on price and quantities per episode of treatment for medicines and supplies required for fast breathing and any PSBI sub-classifications. The price of medicines and consumables used are presented in Table S3 in the **Online Supplementary Document** for seven days of treatment. The average price of antibiotics used was calculated based on the price per size of packets / bottles of medicines, the number of days treated with a given medication and the number of packets / bottles used. While the cost of the injection was calculated per administration, oral amoxicillin was calculated per treatment course. A 60 millilitre (ml) bottle of amoxicillin could be used for six days, therefore, two bottles were required for seven days of treatment and one bottle for one-six days of treatment. Data on quantities and prices for supplies used were estimated based on the usage for the patients treated and were directly collected from each site by interviewing research managers or nodal officers using a structured data collection format. Costs of additional communication material used, and other demand-generating activities were also collected from nodal officers using a structured questionnaire.

### Analysis

Costs were analysed separately for 1) pre-outpatient activities 2) activities related to outpatient treatment 3) operation and administrative costs.

#### 1. Pre-outpatient cost

Costs for pre-outpatient activities were estimated using weighted average costs under identification, assessment, referral, and management of sick young infants using IMNCI (all interventions under domains 1 and 2 in **Table 2** except those who received treatment for fast breathing) and the number of infants receiving that activity. Staff costs for each activity were estimated based on the salary of the providers, and the total duration spent by a provider for that activity. The total time spent per child assessed was dependent on the following three factors. 1) The average time taken by each type of provider for a given activity. 2) The proportion of infants receiving an intervention / activity by each provider type, for example, if the medical officer assessed 80% of infants for an average of 10 minutes, and the nurse assessed 20% of infants for an average of 15 minutes, then the average time by a medical officer will be eight minutes and by a nurse will be three minutes per infant receiving that activity, thus on average, an infant’s assessment would take 11 minutes. 3) The proportion of infants receiving activity from more than one provider during a visit. For example, if during a given visit, both a medical officer and a nurse were providing treatment for five and 10 minutes each, respectively, then the percentage allocation will be 100% for the medical officer and 100% for a nurse, and it would take 15 minutes for an infant during a visit. However, if a medical officer provides treatment to only 80% of infants and a nurse to 90%, the average time per infant treated will be 13 minutes (four minutes for the medical officer and nine minutes for the nurse).

ASHAs were not paid salaries but were paid an incentive of Indian Rupee (₹) 250 (US$3.65) when they completed six or seven postnatal home visits and 50% (Indian ₹125) if they made two-five home visits. Based on the live births who got at least six visits, and those who got two-five visits, total costs for postnatal visits were calculated by assuming that 20% of the costs were attributed to PSBI [24]. Referral costs were estimated based on a transport incentive of Indian ₹100 per referred infant paid to ASHA and the number of infants referred by ASHA for further assessments. If the mother or family presented the child themselves at a primary care centre, then the ASHA did not receive this incentive. The cost of supplies was based on the quantities used, and prices were also added to the identification and referral costs. For consumables, 20% of the costs were attributed to PSBI, except for a mother card, where 100% of costs were estimated. Fifty per cent of the annualised cost of HBNC kits and weighing scales procured were considered for strengthening PSBI identification under non-consumable costs.

#### 2. Outpatient treatment cost

The outpatient treatment costs were calculated per infant treated with “recommended / acceptable” treatment separately for fast breathing and PSBI (including clinical severe infection and severe pneumonia sub-classifications). Total outpatient recurrent costs were estimated per young infant treated by adding costs of staff time, medicines, and consumables per treated child. Weighted average costs were calculated for PSBI depending on the number of infants treated and total costs under each PSBI sub-classification. While the outpatient costs for each PSBI sub-classification were estimated using infants treated with recommended treatment, the costs per follow-up visit were calculated using all treated young infants with fast breathing and PSBI.

#### 3. Operational and administrative cost

Operational costs were estimated per live birth and not per child treated, as these activities benefited all newborns. The costs for administrative staff were calculated based on the full-time equivalent of each staff type (calculated by multiplying the number of staff by the average time spent in a day and the number of days that they spent on PSBI activities in a year) and their salaries. The total costs for the training of the staff were calculated based on per day per trainee costs and the number of staff trained. The per trainee daily costs were estimated using the number of days of training provided, the number of trainees, honorarium for trainers, per diem for trainees, costs of training material and costs for rental of facilities and refreshments. Fifty per cent of the costs reported for IEC and job aids were assigned to PSBI. In addition, in the Palwal district, costs were included for specific activities such as 100% of costs for school rallies and the super village challenge as a special initiative for demand generation, and 10% of the transport per diem paid to ASHAs for attending the supervision meetings during the year.

#### 4. Total cost

Total costs per infant treated were estimated separately for treatment of any PSBI sub-classification and fast breathing by adding the pre-outpatient costs per infant treated, outpatient costs per infant treated, and operational costs per live birth. The operational cost per live birth was calculated by adding the total recurrent costs for staff time, supplies, operations, and administration and dividing it by the total live births at each site.

Local currencies are converted into US$ based on the mid-market exchange rate for July 2018 at 1US$ = Indian ₹68.5 [29].

### Ethical approval

The study was approved by the institutional ethics committees of each participating institution and the WHO Ethics Review Committee. Written informed consent was obtained from caregivers for data collected.

## RESULTS

**Table 3** shows that among the total live births, 87% in Lucknow and 54% in Palwal received at least one postnatal home visit. Of the total infants who received postnatal home visits, 5.9% in Palwal and 5.1% in Lucknow were identified with pneumonia or PSBI. While all infants identified with pneumonia received recommended / acceptable treatment, 59% in Palwal and 40% in Lucknow of those treated for PSBI received recommended / acceptable treatment. The rest received partial or non-compliant treatment.

**Table 3:** Number of live births and infants treated for pneumonia and PSBI with different treatment categories in Palwal and Lucknow, 2018-19*

No	Parameter	Palwal, Haryana, n	Lucknow, Uttar Pradesh, n
1	Live births	4271	13 895
2	Infants received at least one postnatal home visit	2316	12 040
3	Infants identified with pneumonia or PSBI	136	610
4	Infants treated for pneumonia	22	94
5	Infants treated for pneumonia with the recommended treatment	22	94
6	Infants treated for PSBI	114	516
7	Infants treated for PSBI with recommended or acceptable treatment	67	208
8	Infants treated for PSBI with partial treatment or non-compliant treatment	47	308

PSBI – possible serious bacterial infection

*Source: authors’ estimates.

**Table 4** reports the number of visits and young infants covered under different activities; the percentage of visits conducted by different providers for a specific activity and the average time taken for each infant per visit by different providers at both sites. While only 21% of the infants with danger signs were mobilised by ASHA in Palwal, 65% of the infants in Lucknow were brought in by ASHA to primary care facilities for assessment and classification.

**Table 4:** Number of visits or infants, time spent (in minutes) per visit and percentage of visits covered by each provider(s) for each activity in Palwal and Lucknow, 2018-19*

No	Interventions / activities	Palwal, Haryana		Lucknow, Uttar Pradesh	
		**Number of visits or infants receiving intervention**	**Percentage of visits covered by provider type; provider time spent per visit (in minutes)**	**Number of visits or infants receiving intervention**	**Percentage of visits covered by provider type; provider time spent per visit (in minutes)**
**1**	**Identification and referral / mobilisation of sick young infants at the community level**				
1.1	Postnatal home visits for identifying danger signs	13 208	ASHA†: 100%, 20	70 645	ASHA†: 100%, 19
1.2	Infants with PSBI signs that received referral / mobilised by ASHA† to the nearest primary health facility for treatment	33	ASHA†: 100%, 45	685	ASHA†: 100%, 36
**2**	**Management of sick young infants at the facility level using IMNCI protocols**				
2.1	Infants with PSBI^‡^ signs assessed and classified	159	ANM: 10%, 15, nurse: 10%, 3, medical officer: 80%, 9	1049	ANM: 10%, 20, nurse: 10%, 17, medical officer: 80%, 7
2.2	Infants with PSBI^‡^ signs that received counselling and referral advice to higher facilities	135	ANM: 10%, 11, nurse: 10%, 3, medical officer: 80%, 7	640	ANM: 10%, 23, nurse: 10%, 12, medical officer: 80%, 7
2.3	Infants are given pre-referral doses for those who accepted a referral	32	ANM: 10%, 10, nurse: 10%, 5, medical officer: 80%, 6	14	ANM: 10%, 13, nurse: 10%, 8, medical officer: 80%, 8
2.4	Infants 7-59 d old treated for pneumonia	22	ANM: 7%, 7, nurse: 93%, 2, medical officer: 30%, 4	94	ANM: 10%, 10, nurse: 90%, 8, medical officer: 10%, 4
**3**	**Outpatient treatment for PSBI in primary care settings when a referral was not feasible**				
3.1	Infants 0-6 d treated for severe pneumonia	0	Not applicable	Treated, total = 19, recommended = 17	ANM: 10%, 11; nurse: 10%, 9; medical officer: 90%, 4
3.2	Infants treated for clinical severe infection‡	Treated, total = 108, recommended = 67	ANM: 7%, 6; nurse: 93%, 3; medical officer: 93%, 4	Treated, total = 452, recommended = 191	ANM: 10%, 10; nurse: 10%, 7; medical officer: 90%, 4
3.3	Number of follow-up facility visits for a reassessment of the sick young infant	204	ANM: 7%, 15; nurse: 93%, 11; medical officer: 30%, 7	1302	ANM: 10%, 14; nurse: 10%, 10; medical officer: 90%, 6

ASHA – accredited social health activist, PSBI – possible serious bacterial infection, IMNCI – integrated management of newborn and childhood illness, d – days, ANM – auxiliary nurse midwife

*Source: authors’ estimates.

†While the time spent by ASHA and the proportion of the total visits / total time attributed to ASHA have been mentioned in the table, the costs for ASHA were not calculated based on time costs as they are not paid a salary but were given incentives for certain activities.

‡Clinical severe infection: the presence of any of the following signs – movement only on stimulation, stopped feeding well, severe chest in-drawing, the body temperature of> = 38°C or <35.5°C.

Direct intervention costs for staff time, medicines, and consumables were estimated per young infant treated with recommended / acceptable treatment for pneumonia and PSBI sub-classifications (**Table 5**). The direct costs for pneumonia treatment were US$0.6 in Palwal and US$0.9 in Lucknow. The costs of medicines were the same at both sites for pneumonia, as 100% received recommended / acceptable treatment. Direct costs of recommended / acceptable outpatient treatment for any sub-classification of PSBI per young infant treated were US$6 in Palwal and US$6.6 in Lucknow. The staff time costs form a major part of the direct treatment costs. The staff time costs, both in absolute terms as well as per cent of total costs, were higher in Lucknow at 85.3% as compared to 73.6% in Palwal because of the larger time stated per activity and higher average salaries for nurses. The average costs of medicines were the same across both sites. The costs of consumables were lower in Lucknow as compared to Palwal due to the lower price for syringes and needles reported for treatment.

**Table 5:** Direct costs of staff time*, medicines, and consumables per young infant and number of infants receiving recommended / acceptable treatment for fast breathing and different PSBI types, 2018-19†

Costs and numbers receiving treatment under each category	Pneumonia	Severe pneumonia	Clinical severe infection (CSI)	Average direct costs for PSBI
	Palwal, Haryana			
Staff costs (US$)	0.2	NA‡	4.4	4.4
Medicines costs (US$)	0.4	NA‡	0.6	0.6
Consumables costs (US$)	0	NA‡	1.0	1.0
Treated cases (n)	22	0	67	67
	Lucknow, Uttar Pradesh			
Staff (US$)	0.5	6.5	5.6	5.7
Medicines (US$)	0.4	0.6	0.5	0.6
Consumables (US$)	0.0	0.5	0.4	0.4
Treated cases (n)	94	17	191	208

CSI – clinical severe infection, PSBI – possible serious bacterial infection, US$ – US dollar, NA – not applicable

*Staff time costs across different treatment categories do not include costs of assessment and follow up.

†Source: Authors’ estimates.

‡Not applicable as no infant received treatment for severe pneumonia in Haryana.

The direct costs of recommended / acceptable treatment estimated per treated young infant and indirect costs estimated per live birth are shown in **Table 6**. The average total costs for recommended/ acceptable treatment per infant treated for PSBI was estimated at US$15.9 (Palwal US$17.2 and Lucknow US$15.4) for PSBI and US$ $10.1 (Palwal US$11.8 and Lucknow US$9.7) for pneumonia. The pre-outpatient costs per treated infant were estimated to be around 29% (US$5.1 in Palwal and US$4.5 in Lucknow) of the total PSBI direct and indirect costs. The higher pre-outpatient costs in Palwal were due to a higher percentage (39%) of young infants receiving six-seven postnatal visits compared to 17% in Lucknow, which implied greater incentive payments for ASHA. The total referral costs, which is also a part of the pre-outpatient treatment, were lower in Palwal than in Lucknow, due to the lower number of infants referred by ASHA (33 in Palwal vs. 685 in Lucknow) but remained the same per infant across the two sites at US$1.5 per infant referred. The pre-outpatient costs were added to outpatient treatment and follow-up costs to estimate the direct recommended / acceptable treatment costs per infant treated for PSBI at US$13.0 in Palwal and US$13.6 in Lucknow.

**Table 6:** Pre-outpatient, outpatient, operational, administrative, and total costs for fast breathing and PSBI for recommended / acceptable treatment at two sites in India, 2018-19*

No	Cost heads	Palwal, Haryana (US$)	Lucknow, Uttar Pradesh (US$)
**1**	**Direct Interventions / activity costs heads**		
1.1	Costs for pre-outpatient treatment activities	5.1	4.5
1.2	Outpatient treatment costs (staff + medicines + consumables) per infant for fast breathing	0.6	0.9
1.3	Outpatient treatment costs (staff time + medicines + consumables) per infant for any PSBI type	6.0	6.6
1.4	Costs of follow-up assessments	1.8	2.5
1.5	Total direct intervention costs per infant treated for pneumonia	7.5	7.9
1.6	Total direct intervention costs per infant treated for PSBI	13.0	13.6
**2**	**Indirect operational costs**		
2.1	Operational costs	1.6	0.6
2.2	Administrative costs of staff	2.6	1.2
2.3	Total operational and administrative costs per live birth	4.3	1.8
**3**	**Total**† **and incremental costs (direct + indirect)**		
3.1	Total costs (1.6 + 2.3) per treated infant for PSBI	17.2	15.4
3.2	Total costs (1.5 + 2.3) per treated infant for pneumonia	11.8	9.7
3.3	Incremental costs excluding staff costs for PSBI	6.1	4.3
3.4	Incremental costs excluding staff costs for pneumonia	3.5	2.2
3.5	Total costs for PSBI or pneumonia per live birth	5.1	2.7
3.6	Pre-outpatient costs % total costs per treated young infant with PSBI	29.8%	29.2%
3.7	Operation and administrative costs % total costs per treated young infant with PSBI	24.7%	11.9%

US$ – US dollar, PSBI – possible serious bacterial infection

*Source: Authors’ estimates.

†The total costs may be slightly different from the sums due to rounding errors.

The operational costs (including communication, demand generation activities and training) and administrative costs were 25% in Palwal compared to 12% in Lucknow of the total costs of treating PSBI. The incremental costs, excluding staff costs, were US$6.1 and US$4.3 for PSBI, and US$3.5 and US$2.2 in Palwal and Lucknow, respectively. This implies the share of staff costs in the total (direct + indirect) costs of treating PSBI with recommended treatment was 64% in Palwal and 72% in Lucknow. The larger operation costs in Palwal were due to communication costs for the demand generation activities and transport incentives paid for supervisory visits, and higher staff costs in Lucknow were due to higher salaries and longer time reported per activity. Costs per live birth were US$5 in Palwal compared to US$3 in Lucknow.

## DISCUSSION

### Main results and their interpretations

The weighted average costs across both sites for treating young infants with PSBI in an outpatient setting with recommended / acceptable treatment was US$15.9 (95% CI = US$15.4-16.3) considering all costs, i.e., the cost for identification, postnatal home visits, referrals, treatment, daily assessments, operation, and administration. The corresponding costs for treating pneumonia were US$10.1 (95% CI = US$9.7-10.6). Additionally, in a population of 4271 annual live births in the study area of Palwal and 13895 in Lucknow, investment for management and treatment of PSBI or pneumonia was estimated at an average cost of US$5 per live birth in Palwal and US$3 per live birth in Lucknow. Our results show that recommended direct treatment costs, including pre-outpatient and follow-up assessments for PSBI, were US$13.0 in Palwal and US$13.6 in Lucknow. For pneumonia, the direct costs of treatment with oral amoxicillin were US$7.5 in Palwal and US$7.9 in Lucknow. Low direct costs for pneumonia are primarily due to low staff time costs (mothers are trained to give the antibiotic to the infant) and no consumable costs.

While the total costs at the two sites are a bit different per treated infant, with variations in components of direct costs and operational costs, the direct costs of treatment were higher in Lucknow because of higher staff (higher salaries and more time spent on activities) costs. In Palwal, there were more contractual nurses, who had lower average salaries than regular staff. The pre-outpatient costs per treated infant for the identification of sick infants, and their subsequent management were more than a quarter of the total costs at both sites. The WHO and the Government of India both recommend postnatal home visits to improve maternal and newborn survival [26,27,30,31]. However, it is important to convert these postnatal home visits into successful referrals for those with danger signs. Despite a higher percentage (5.9% in Palwal and 5.1% in Lucknow) of infants identified with pneumonia or PSBI (from those who received postnatal home visits) and a larger percentage of complete postnatal home visits (among total postnatal visits) in Palwal, mobilisation by ASHA was only 21% in Palwal and 65% in Lucknow of those assessed for danger signs in public facilities. Pre-outpatient costs were higher in Palwal (US$5.1) than Lucknow (US$4.5) due to the larger number of complete postnatal home visits, but it did not lead to successful mobilisation by ASHA. This led Palwal to use additional social mobilisation techniques, which led to higher operating costs for Palwal. It is important to note that, using community mobilisation strategies, the Palwal site was able to demonstrate a higher coverage (70%) [18] compared to the Lucknow site (53%) [19], which translated to the empowerment of families to access timely and appropriate health care.

Marginal operational and administrative costs of staff for management, supervision, training, and communications, are an important component of the effective implementation of the program. These formed 24% of the total costs for recommended treatment in Palwal and 12% in Lucknow. The share and absolute costs were higher at the Palwal site, as more was spent on communication, demand generation activities, and the staff implementing these activities. The lower costs per live birth in Lucknow were due to lower operational and administrative costs serving a larger number of live births.

Our results showed that staff costs were one of the major components of costs for treatment. These comprised 64% and 72% of the total costs of treatment for PSBI in Palwal and Lucknow, respectively. The incremental costs (excluding staff time costs from total costs) were US$6.1 in Palwal and US$4.3 in Lucknow per infant receiving recommended/ acceptable treatment for PSBI. These incremental costs were US$3.5 and US$2.2 for pneumonia in Palwal and Lucknow, respectively.

In summary, while pre-outpatient costs for mobilisation may be important, the costs paid in the form of ASHA incentives may not imply better mobilisation. While mobilisation by ASHA worked in the case of Lucknow, Palwal had to implement additional social mobilisation methods to educate mothers to identify danger signs and bring the infant to the primary care facility, which led to higher operating costs for them. This might have important national policy-level implications on strategies to improve coverage while implementing PSBI management guidelines in similar settings.

### Comparison with past studies

Our results show lower costs for both pneumonia and PSBI, as we did not include the costs of antenatal care and treatment of mild infections for non-PSBI cases, and restricted the assessment costs only for the infants identified with PSBI. Further, as this was the implementation research, a large part of the supplies for pre-outpatient and outpatient treatment, and operational costs were considered health system costs and were not included. Our results show costs to be lower than those found in the AFRINEST study for three African countries [24], and in Ethiopia [22], for management of PSBI at the community level or the health post level, respectively. The cost per mother / baby in Ethiopia for community management of PSBI was estimated at US$1.78 for a 100 000 population in routine set-up, with 95% of women receiving at least four visits. The costs (including the opportunity costs of the providers) of clinical severe infection in an outpatient setting were estimated at US$37 in the Ethiopian study (2015 US$) [22] and US$ 32.3 for the most cost-effective intervention (injectable gentamicin plus oral amoxicillin for seven days) in the AFRINEST study [24].

The average costs of treating pneumonia across the two sites were less than half (US$10.1) compared to US$26.2 in the AFRINEST study in an outpatient setting [24]. A study from Pakistan showed household costs (direct and indirect including costs of medicines provided by Lady health worker) of chest indrawing pneumonia management in children 2-59 months old as US$1.5 in community ambulatory care and US$7.9 in outpatient care in 2013 US$ [32]. Zhang et al. reported that the cost per episode in 2013 US$ for the management of chest indrawing pneumonia in children was US$4.3 in the community, US$51.7 in outpatient facilities and US$242.7 to US$559.4 at different levels of hospital for inpatient settings in low and middle-income countries [23].

The WHO recommended outpatient treatment when a referral is not feasible for PSBI was reportedly cost-effective at US$32.3 in the AFRINEST study [24]. In our study, the average cost of strengthening this recommended treatment at US$ 16.0 for PSBI is therefore quite cost-effective. Manandhar et al. argue that an intervention that costs less than US$127 (World Bank figure) is cost-effective [33]. Black et al. suggested that “interventions costing less than per capita gross national income per disability-adjusted life year averted can be termed “very cost-effective,” and those costing less than three times per capita gross national income can be termed “cost-effective” [34].

### Strengths and weaknesses

Our study has strength in terms of estimating the true costs of scaling up the existing program. This implementation research was conducted in a program setting, and both average and incremental costs were estimated based on actual implementation costs incurred for treatment and strengthening of the program. The estimated average costs are marginal in the sense that these did not include the capital and equipment costs, which were considered part of the health system. Further, only additional time for the staff for activities and fractional costs of certain consumables and non-consumables, considered important for strengthening health systems for additional infants treated, were included. Operation costs- communication, demand generation and training were only considered for system strengthening. Scaling up at these costs is only possible if the health system continues to function at least in its current state. We did not estimate societal or household costs. We also were unable to estimate the cost-effectiveness in this study, because we did not have pre-intervention coverage or mortality data, and the scope of the study was to estimate costs for budgeting purposes and provide policymakers guidelines to strengthen the PSBI management in outpatient settings. The cost data have been presented for 2018-19, the year for which the data was collected. However, in 2022, when adjusted for inflation, the average costs per young infant treated for PSBI and pneumonia would be US$18.5 and US$11.7, respectively.

## CONCLUSIONS

Outpatient treatment is not only beneficial in terms of reduced costs but is also less disruptive for the families and has less risk of getting hospital-acquired infections [35,36]. Outpatient management is cost-effective using a combination of injection gentamicin plus oral amoxicillin for PSBI when a referral is not feasible, and oral amoxicillin for those with only fast breathing. Successful mobilisation of infants with PSBI is important for treatment, which can be achieved through a combination of different methods – postnatal home visits by ASHA for the identification and referral by ASHA as in Lucknow; or successful mobilisation by mothers by educating them through social mobilisation activities as in case of Palwal. This has cost implications on different components. Community mobilisation interventions, strengthening the skills of health workers, and prompt care seeking from appropriate health care providers are critical for the appropriate management of sick young infants. Investments in the programmatic functions such as training of the providers, continuous communication and visits for follow-up, demand generation through mothers and ASHA, supervision and administration are essential for the successful implementation. In short, when a referral is not feasible, the costs of activities for the successful identification, prompt care seeking and timely initiation of outpatient management of young infants with PSBI with recommended antibiotic regimens and follow-up are reasonable and must be budgeted. This will reduce the mortality from neonatal sepsis, one of the leading causes of neonatal deaths.

## Additional material


Online Supplementary Document

## References

[1] GBD 2013 Mortality and Causes of Death Collaborators Global, regional, and national age-sex specific all-cause and cause-specific mortality for 240 causes of death, 1990–2013: a systematic analysis for the Global Burden of Disease Study 2013. Lancet. 2015;385:117-71. 10.1016/S0140-6736(14)61682-2 25530442 PMC4340604

[2] Murthy S Godinho MA Guddattu V Lewis LES Nair NS Risk factors of neonatal sepsis in India: A systematic review and meta-analysis. PLoS One. 2019;14:e0215683. 10.1371/journal.pone.0215683 31022223 PMC6483350

[3] United Nations Inter-agency Group for Child Mortality Estimation (UN IGME). Levels and Trends in Child Mortality: Report 2021. New York: United Nations Children’s Fund; 2021. Available: https://data.unicef.org/resources/levels-and-trends-in-child-mortality/. Accessed: 6 Sep 2022.

[4] Ministry of Health and Family Welfare, Government of India. National Family Health Survey (NFHS-5) 2019-21: Compendium of fact sheets. Key indicators for 14 States/UTs (Phase 2). Available: https://data.unicef.org/resources/levels-and-trends-in-child-mortality/. Accessed:15 September 2022.

[5] World Health Organization. Pocket Book of Hospital Care for Children: Guidelines for the Management of Common Childhood Illnesses. 2nd edition. Geneva: World Health Organization; 2013. 24006557

[6] World Health Organization. Guideline. Managing possible serious bacterial infection in young infants when referral is not feasible. Geneva 2015. Available: https://www.who.int/publications/i/item/9789241509268. Accessed: 15 September 2022. 26447263

[7] Kozuki N Guenther T Vaz L Moran A Soofi SB Kayemba CN A systematic review of community-to-facility neonatal referral completion rates in Africa and Asia. BMC Public Health. 2015;15:989. 10.1186/s12889-015-2330-0 26419934 PMC4589085

[8] Bang AT Bang RA Baitule SB Reddy MH Deshmukh MD Effect of home-based neonatal care and management of sepsis on neonatal mortality: field trial in rural India. Lancet. 1999;354:1955-61. 10.1016/S0140-6736(99)03046-9 10622298

[9] Zaidi AKM Tikmani SS Warraich HJ Darmstadt GL Bhutta ZA Sultana S Community-based treatment of serious bacterial infections in newborns and young infants: a randomized controlled trial assessing three antibiotic regimens. Pediatr Infect Dis J. 2012;31:667-72. 10.1097/INF.0b013e318256f86c 22481421

[10] Baqui AH Saha S Ahmed A Shahidulla M Quasem I Roth DE Safety and efficacy of alternative antibiotic regimens compared with 7 day injectable procaine benzylpenicillin and gentamicin for outpatient treatment of neonates and young infants with clinical signs of severe infection when referral is not possible: a randomised open label, equivalence trial. Lancet Glob Health. 2015;3:e279-87. 10.1016/S2214-109X(14)70347-X 25841891

[11] Mir F Nisar I Tikmani SS Baloch B Shakoor S Jehan F Simplified antibiotic regimens for treatment of clinical severe infection in the outpatient setting when referral is not possible for young infants in Pakistan (Simplified Antibiotic Therapy Trial [SATT]): a randomised, open-label, equivalence trial. Lancet Glob Health. 2017;5:e177-85. 10.1016/S2214-109X(16)30335-7 27988146 PMC5250591

[12] African Neonatal Sepsis Trial (AFRINEST) Group Tshefu A Lokangaka A Ngaima S Engman C Esamai F Simplified antibiotic regimens compared with injectable procaine benzylpenicillin plus gentamicin for treatment of neonates and young Infants with clinical signs of severe infection when referral is not possible: a randomized equivalence trial. Lancet. 2015;385:1767-76. 10.1016/S0140-6736(14)62284-4 25842221

[13] African Neonatal Sepsis Trial (AFRINEST) group Tshefu A Lokangaka A Ngaima S Engmann C Esamai F Gisore P Oral amoxicillin compared with injectable procaine benzylpenicillin plus gentamicin for treatment of neonates and young infants with fast breathing when referral is not possible: a randomised, open-label, equivalence trial. Lancet. 2015;385:1758-66. 10.1016/S0140-6736(14)62285-6 25842223

[14] Ministry of Health and Family Welfare, Guide for Orientation of Primary Health Care Providers in Management of Sepsis in Young Infants, National Health Mission, Child Health Division, Government of India, June 2017.

[15] Ministry of Health and Family Welfare. Government of India. Use of gentamicin by ANMs for management of sepsis in young infants under specific situations. Operational Guidelines. 2014. Available: https://www.healthynewbornnetwork.org/hnn-content/uploads/Operational-Guidelines_India.pdf. Accessed: 16 May 2020.

[16] Government of India. Addendum to Operational Guidelines on Use of Gentamicin by ANMs for Management of Sepsis in Young Infants under Specific Situations. Ministry of Health and Family Welfare Government of India; New Delhi: 2018 Available: https://nhm.gov.in/New_Updates_2018/Om_and_orders/rmncha/child_health/Addendum.pdf. Accessed: 25 June 2020.

[17] Nisar YB Aboubaker S Arifeen SE Ariff S Arora N Awasthi S A multi-country implementation research initiative to jump-start scale-up of outpatient management of possible serious bacterial infections (PSBI) when a referral is not feasible: Summary findings and implications for programs. PLoS One. 2022;17:e0269524. 10.1371/journal.pone.0269524 35696401 PMC9191694

[18] Mukhopadhyay R Arora NK Sharma PK Dalpath S Limbu P Kataria G Lessons from implementation research on community management of Possible Serious Bacterial Infection (PSBI) in young infants (0-59 days), when the referral is not feasible in Palwal district of Haryana, India. PLoS One. 2021;16:e0252700. 10.1371/journal.pone.0252700 34234352 PMC8279773

[19] Awasthi S Kesarwani N Verma RK Agarwal GG Tewari LS Mishra RK Identification and management of young infants with possible serious bacterial infection where referral was not feasible in rural Lucknow district of Uttar Pradesh, India: An implementation research. PLoS One. 2020;15:e0234212. 10.1371/journal.pone.0234212 32497092 PMC7272098

[20] Goyal N Rongsen-Chandola T Sood M Sinha B Kumar A Qazi SA Management of possible serious bacterial infection in young infants closer to home when referral is not feasible: Lessons from implementation research in Himachal Pradesh, India. PLoS One. 2020;15:e0243724. 10.1371/journal.pone.0243724 33351810 PMC7755274

[21] Roy S Patil R Apte A Thibe K Dhongade A Pawar B Feasibility of implementation of simplified management of young infants with possible serious bacterial infection when referral is not feasible in tribal areas of Pune district, Maharashtra, India. PLoS One. 2020;15:e0236355. 10.1371/journal.pone.0236355 32833993 PMC7446882

[22] Mathewos B Owen H Sitrin D Cousens S Degefie T Wall S Community-Based Interventions for Newborns in Ethiopia (COMBINE): Cost-effectiveness Analysis. Health Policy Plan. 2017;32:i21-32. 10.1093/heapol/czx054 28981760

[23] Zhang S Sammon PM King I Ana LA Andrade L Toscano CA Cost of management of severe pneumonia in young children: systematic analysis. J Glob Health. 2016;6:010408. 10.7189/jogh.06.010408 27231544 PMC4871066

[24] Garg CC Tshefu A Longombe AL Kila J-SN Esamai F Gisore P Costs and cost-effectiveness of management of possible serious bacterial infections in young infants in outpatient settings when referral to a hospital was not possible: Results from randomized trials in Africa. PLoS One. 2021;16:e0247977. 10.1371/journal.pone.0247977 33720960 PMC7959374

[25] Government of India. Janani Suraksha Yojana: Features and Frequently Asked Questions. Ministry of Health and family Welfare, Maternal Health Division. New Delhi; 2006. Available: https://nhm.gov.in/WriteReadData/l892s/97827133331523438951.pdf Accessed: 25 June 2020.

[26] Government of India. Home Based Newborn Care Operational guidelines. Ministry of Health and Family Welfare. New Delhi, 2014. Available: https://www.healthynewbornnetwork.org/hnn-content/uploads/Revised_Home_Based_New_Born_Care_Operational_Guidelines_2014.pdf. Accessed: 25 June 2020.

[27] Government of India. In service Integrated Management of Neonatal and Childhood management (Module 1 to Module 9). Ministry of Health and Family Welfare, New Delhi; 2009. Available: http://www.sihfw.nrhmharyana.gov.in/Modules/Child%20Health/IMNCI/In%20Services%20IMNCI/imnci_cover_page.pdf. Accessed: 25 June 2020.

[28] Hendrich A Chow MP Skierczynski BA Lu Z A 36-hospital time and motion study: how do medical-surgical nurses spend their time? Perm J. 2008;12:25-34. 10.7812/tpp/08-021 21331207 PMC3037121

[29] Currency Table. USD – US Dollar. Available: https://www.xe.com/currencytables/?from=USD&date=2018-07-31. Accessed: 25 June 2023.

[30] World Health Organization. WHO recommendations on postnatal care of the mother and newborn.2013. Available: https://www.who.int/docs/default-source/mca-documents/nbh/brief-postnatal-care-for-mothers-and-newborns-highlights-from-the-who-2013-guidelines.pdf. Accessed: 25 June 2020. 24624481

[31] World Health Organization. Integrated Management of Childhood Illness (IMCI), chart booklet. Geneva: WHO; 2014. Available: http://apps.who.int/iris/ bitstream/10665/104772/16/9789241506823_Chartbook_eng.pdf. Accessed: 25 June 2020.

[32] Sadruddin S Shehzad S Bari A Khan A Khan A Qazi S Household costs for treatment of severe pneumonia in Pakistan. Am J Trop Med Hyg. 2012;87:137-43. 10.4269/ajtmh.2012.12-0242 23136289 PMC3748514

[33] Manandhar DS Osrin D Shrestha BP Mesko N Morrison J Tumbahangphe KM Effect of a participatory intervention with women’s groups on birth outcomes in Nepal: cluster-randomised controlled trial. Lancet. 2004;364:970-9. 10.1016/S0140-6736(04)17021-9 15364188

[34] Black RE, Laxminarayan R, Temmerman M, Walker N, editors. 2016. Reproductive, Maternal, Newborn, and Child Health. Disease Control Priorities, third edition, volume 2. Washington, DC: World Bank. License: Creative Commons Attribution CC BY 3.0 IGO. 27227205

[35] Laxminarayan R Duse A Wattal C Zaidi AK Wertheim HF Sumpradit N Antibiotic resistance-the need for global solutions. Lancet Infect Dis. 2013;13:1057-98. 10.1016/S1473-3099(13)70318-9 24252483

[36] Mathur P Malpiedi P Walia K Srikantiah P Gupta S Lohiya A Indian Healthcare Associated Infection Surveillance Network collaborators Health-care-associated bloodstream and urinary tract infections in a network of hospitals in India: a multicentre, hospital-based, prospective surveillance study. Lancet Glob Health. 2022;10:e1317-25. 10.1016/S2214-109X(22)00274-1 35961355

