# Supplementary material for: Cost of treating sick young infants (0-59 days) with Possible Serious Bacterial Infection in resource-constrained outpatient primary care facilities: An insight from implementation research in two districts of Haryana and Uttar Pradesh (India)
Source: J Glob Health. 2023 Aug 18;13:04062. doi: 10.7189/jogh.13.04062 (PMC10436679; doi:10.7189/jogh.13.04062)
Supplement: Online Supplementary Document [file jogh-13-04062-s001.pdf]

Supplementary material

Supplementary Table 1: Number of observations from different providers from which time data was collected for activities using both self-observed and observed by the third party by facility type

	Palwal, Haryana				Lucknow			
	Subcenter	PHC	CHC	Total	Subcenter	PHC	CHC	Total
Medical Officers	NA	1	24	26	NA	22	26	48
Nurses	NA		2	2	NA	1	14	15
ANM	6	2	2	10	49	5	1	55

Source: Authors' estimates

Note: PHC: Primary health centers; CHC: Community Health Centers; NA: not applicable

Supplementary Table 2: Average annual salary of the providers

I	Average annual Salary in US$ for				
I.1	Medical officer		13504		13796
I.2	Nurse		5956		8597
I.3	ANM		6131		6123

Supplementary Table 3: Price of medicines and Consumable Supplies for 7 days treatment in US $ by sites

	Palwal, Haryana	Lucknow, Uttar Pradesh
**Medicines**		
Oral amoxicillin (powder) (125 mg/5 ml) 60 ml bottle - cost per 7 days treatment	0.43	0.43
Gentamicin injection (liquid) (40 mg/ml) - 2 ml ampoule - cost per administration)	0.2	0.2
**Consumable Supplies**		
For gentamicin administration for 7 days : 7 Injections (1ml syringe and 1 needle 26 gauge) + 14 ml spirit + 50 gm cotton)	1.1	0.66

Source: Authors' estimates
